# BowStrap v1.0: Assigning statistical significance to expressed genes using short-read transcriptome data

**DOI:** 10.1186/1756-0500-5-275

**Published:** 2012-06-07

**Authors:** Peter E Larsen, Frank R Collart

**Affiliations:** 1 Biosciences Division, Argonne National Laboratory, Lemont, IL, 60490, USA

**Keywords:** Transcriptomics, Next Generation Sequencing, Gene expression, Metatranscriptome

## Abstract

**Background:**

Background: Deep RNA sequencing, the application of Next Generation sequencing technology to generate a comprehensive profile of the message RNA present in a set of biological samples, provides unprecedented resolution into the molecular foundations of biological processes. By aligning short read RNA sequence data to a set of gene models, expression patterns for all of the genes and gene variants in a biological sample can be calculated. However, accurate determination of gene model expression from deep RNA sequencing is hindered by the presence of ambiguously aligning short read sequences.

**Findings:**

BowStrap, a program for implementing the sequence alignment tool ‘Bowtie’ in a bootstrap-style approach, accommodates multiply-aligning short read sequences and reports gene model expression as an averaged aligned reads per Kb of gene model sequence per million aligned deep RNA sequence reads with a confidence interval, suitable for calculating statistical significance of presence/absence of detected gene model expression. BowStrap v1.0 was validated against a simulated metatranscriptome. Results were compared with two alternate ‘Bowtie’-based calculations of gene model expression. BowStrap is better at accurately identifying expressed gene models in a dataset and provides a more accurate estimate of gene model expression level than methods that do not incorporate a boot-strap style approach.

**Conclusions:**

BowStrap v1.0 is superior in ability to detect significant gene model expression and calculate accurate determination of gene model expression levels compared to other alignment-based methods of determining patterns of gene expression. BowStrap v1.0 also can utilize multiple processors as has decreased run time compared to the previous version, BowStrap 0.5. We anticipate that BowStrap will be a highly useful addition to the available set of Next Generation RNA sequence analysis tools.

## Findings

### Background

Deep RNA sequencing (RNAseq) is a powerful tool for assessing gene expression patterns. As Next Generation Sequencing (NGS) becomes an increasingly prevalent tool for scientific investigation, efficient and robust methods for interpreting short read sequence data as gene model expression levels are required. Gene expression is commonly represented as the number of short sequence Reads aligned Per Kilobase of gene sequence per Million of aligned reads (RPKM). However, a significant fraction of the total reads in an RNA-seq experiment cannot be aligned to a unique location in the gene set [1-3]. Accounting for these ambiguously aligning reads is required for an accurate measure of gene expression, especially for gene models with high sequence identity in a transcriptome.

One method used to accommodate multiply-aligning reads is to discard them and consider only those reads that can be aligned to a unique location in gene models or genome [4,5]. ERANGE assigns multiply-aligned reads to gene models according to the distribution frequency of uniquely aligned reads [1]. Cufflinks aligns short reads to genomic sequence, essentially preforming gene discovery analysis with every experimental sequence [6]. While thorough and capable of detecting subtle gene splice variants in expression data, Cufflinks requires very deep sequencing of transcriptomes. Although each of these methods represents gene model expression levels as a measure of number of short sequences that align, none of these approaches provide a measure of the statistical significance for presence or absence of gene model expression nor are these methods expressly designed to distinguish between expression levels of high sequence identity gene models.

BowStrap, presented in an early version in [7], follows a unique approach. BowStrap calculates the expression of gene models as a statistical distribution. Utilization of BowStrap requires a high quality set of gene models and output of the ultra-fast sequence alignment program “Bowtie” [8]. BowStrap uses the output from Bowtie, a file of gene model names and lengths (in base pairs), and the number of desired bootstrap iterations. BowStrap calculates PRKM values across multiple iterations. For each iteration, if an RNAseq read can be aligned to more than one possible location, that read is uniformly assigned to a randomly selected alignment location by BowStrap. This iterative, bootstrap-style approach is the a straightforward way of assigning standard errors and confidence intervals to gene model expression, as opposed to an estimate of read distribution which can provide the expected number of reads that align to a gene model, but is less able to express that value as a distribution that is a function of the specific properties of an RNAseq dataset and particular combination of potentially ‘cross-hybridizing’ gene models. For every gene model in the set, BowStrap reports the RPKM for uniquely aligning reads, and the average and standard deviation for boot-strapped RPKM. A statistical measurement to determine if expression of a gene model has been detected at significant levels can be calculated as a Cumulative Normal Distribution (CND) and expressed as a p-value. The updated and much improved version of BowStrap presented here is faster, capable of running on multiple processors, and includes the very important information for uniquely aligning reads for each gene model. Counts for uniquely aligning reads can be used to further distinguish gene expression levels between highly homologous genes in a transcriptome. To demonstrate the utility of BowStrap relative to other ‘Bowtie’-based methods of calculating gene model expression levels, we compare BowStrap results to gene model expression calculated using ‘Bowtie’s random allocation of multiply-aligned reads and gene model expression calculated by disregarding of multiply aligned reads.

### BowStrap method

The protocol for using BowStrap is as follows:

**Step 1.** Align short read sequence data to gene models using Bowtie, reporting all alignments, i.e.:

$ ./bowtie --all < bowtie_build > <short_reads.fq > <bowtie_output_file>

Where **bowtie_build** is the build file for the set of gene models, short_reads.fq is the file or collection of files of short read sequence data, and **bowtie_output_file** the user-selected file name for Bowtie output.

**Step 2.** Generate resampled gene model expression data using BowStrap, i.e.:

$ perl BowStrap_v1.0.pl < Gene_size_file > <iterations > [<num_processors>] < bowtie_output_file > <output_file>

Where **Gene_size_file** is a tab-separated file of gene model names and gene model sizes, iterations is a user-selected number of boot-strap iterations, **num_processors**, an option only in multi-threaded BowStrap, is a user selected integer for number of processors to be devoted to calculations, **bowtie_output_file** is the output from step 1, and **output_file** is the location for saved results.

The output of BowStrap lends itself to calculation of statistical significance of detected gene model expressed, calculated CND (**Eq. 1**)

(1) $$\text{CND pVal}=1-\int_{-\infty }^{0}\frac{1}{\sqrt{2\pi }\sigma }e^{-\left(\frac{{\left(x-\mu \right)}^{2}}{2\sigma^{2}}\right)}$$

where μ is the average and σ is the standard deviation of a re-sampled gene model’s RPKM. As this equation will fail to return a value if the average or standard deviation is equal to 0, if the average is equal to 0, then the CND pValue is set equal to 1, else if standard deviation is equal to 0, then the CND p-value is set equal to 0. A CND p-value close to zero indicates statistically significant levels of detected gene expression.

### Test BowStrap performance relative to other “bowtie”-based methods

In order to validate BowStrap and compare results to those of other ‘Bowtie’-based methods of gene model expression calculation, we generated a single synthetic RNAseq dataset.

#### Generate a synthetic RNA-seq dataset

Complex synthetic RNAseq read sets were generated to highlight the advantages of the BowStrap approach. This synthetic data set is derived from the combined set of gene models, publically available from the Joint Genome Institute (http://genome.jgi.doe.gov/), for the fungus *Laccaria bicolor* and the plant *Populus tremuloides* (68164 total gene models). The combination of gene sequences from different species complicates the ability to find unique alignments for short sequences. In addition, *P. tremuloides* contains multiple duplicate genes [9] and *L. bicolor* has a high intron density containing multiple exons as short as 7 bp [10] making alignment to genomic sequence with RNA reads more difficult. To insure the synthetic dataset represent a biologically relevant expression pattern, the relative gene model expression values were taken form from a previously published experiment investigating symbiotic interaction between the fungus and plant [11]. Synthetic reads were randomly generated from gene model sequences and used to generate sets of 50, 25, 10, 1, and 0.5 million (M) total 46-mer reads. The results of this were five synthetic metatranscriptomes with the same pattern of relative gene expression levels, but different total amounts of sequence data.

Bowtie alignments indicate that about one third of all synthetic reads do not uniquely align to gene model sequences in these synthesized datasets, highlighting the need for tools like BowStrap in determination of gene expression data from deep RNAseq data.

#### Generate gene model expression values using synthetic RNA-seq data set

In addition to BowStrap method, two other, non-bootstrapped ‘Bowtie’-based methods that use alignments to gene models were used to calculate expression levels. The first uses the default ‘Bowtie’ setting, which randomly assigns reads with multiple possible alignment positions to one of the possible locations. The second uses the ‘Bowtie’ setting for discarding all ambiguously aligning reads, reporting only those reads that align to a single location. BowStrap CND p-values were further adjusted using Benjamini-Hochberg false discovery rate correction [12].

#### Compare BowStrap with alternate methods

For each simulated data set, the total number of significantly expressed gene models was identified (Table 1). For BowStrap, (Benjamini-Hochburg False Discovery Rate corrected CBD p-value < 0.05) were calculated. For alternate ‘Bowtie’-based methods, which do not return a statistical significance with determination of gene model expression, expression was considered to be any RPKM greater than zero. This difference in how expressed gene models are in deified by different methods is an important caveat to keep in mind when comparing results between approaches. Accuracy was determined using significantly expressed gene determination and non-zero expression in synthetic data sets. False negatives, gene models incorrectly detected as expressed, was also calculated. Random assignment of ambiguously aligning reads provides good accuracy, and this method is more accurate than BowStrap for dataset sizes less than 25 M reads. Using randomly assigned reads however results in a far higher proportion of false positives in the detected of expressed genes. While using only uniquely aligning reason average yields the worst accuracy, this method did not produce any false positives. Results indicate that BowStrap approach is highly accurate (>90% accuracy) with at least 10 million reads and is tolerably accurate (74%) in calculating gene expression levels even with very small (i.e. 1 million reads) datasets. There are very few false-positives in the set of significantly expressed gene models, never exceeding 0.5% in 50 M read data set.

**Table 1:** Detection of gene expression

**Dataset**	**Accuracy**			**%False positive**		
	**Random**	**Unique**	**BowStrap**	**Random**	**Unique**	**BowStrap**
**50 M**	0.97	0.91	0.98	2.70	0.0	0.43
**25 M**	0.97	0.91	0.97	2.67	0.0	0.21
**10 M**	0.97	0.90	0.92	2.72	0.0	0.08
**1 M**	0.97	0.88	0.74	3.44	0.0	0.00
**0.5 M**	0.97	0.87	0.66	3.40	0.0	0.00

Three ‘Bowtie’-based methods for gene model expression were considered, using gene model expression values from synthetic RNAseq data of 50, 25, 10, 1, and 0.5 million total 46-mer sequence reads. “Random” uses ‘Bowtie’s default random assignment of multiply aligning reads. “Unique” uses only reads with a single, unambiguous alignment location. “Accuracy” is the proportion of gene models correctly identified as either expressed or absent. For “Random” and “Unique”, detection of expression is defined as an RPKM value greater than 0. For “BowStrap”, detection of expression is defined as Benjamini-Hochburg corrected p-value < 0.05. “% False Positive” is the percent of gene models identified as expressed that are absent in synthetic RNAseq data.

**Figure 1:**
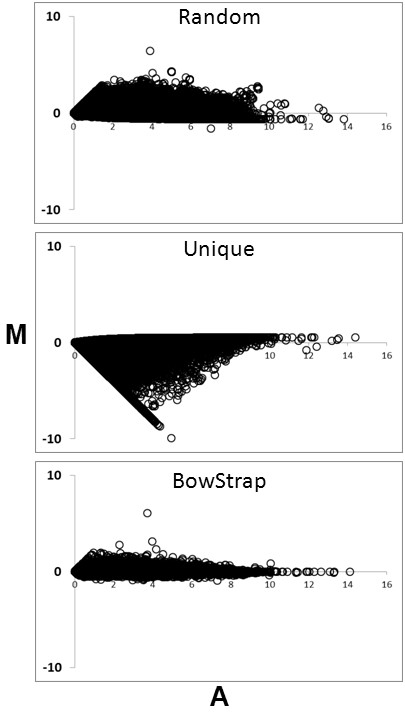
**MA Plots for different gene expression calculation methods.** The 25 M read dataset was selected for this figure. Results for other size datasets are similar. ‘M’ is the log_2_ of calculated gene model expression level divided by known gene model expression level. ‘A’ is the log_2_ of average between calculated and known gene model expression level. Each point in scatter plot is result for a single gene model.

To determine how well different approaches compare at accurately calculating gene model expression levels, we used Spearman’s Rank Correlation between calculated and known gene model expression values for all approaches. BowStrap method is consistently best at calculating gene model expression levels than alternative approaches at every dataset size. (Table 2). To visualize how well calculated gene model expression matches the known gene model expression, MA-plots of the 25 M sequence data set were employed (Figure 1). In an MA plot, perfect correspondence between calculated and known expression levels would result in all points in scatterplot falling on the x-axis. The further points are distributed from the x-axis, the less good is the correspondence between calculated and known gene model expression levels. In this figure, BowStrap shows the closest relationship between calculated and observed gene model expression levels. Using randomly assigned ambiguously aligned reads method tends to over-report gene model expression. Using only uniquely aligned reads performed the worst, most often under-reporting gene model expression levels.

**Table 2:** Spearman’s correlation between calculated gene expression and known gene model expression

**Dataset**	**Random**	**Unique**	**BowStrap**
**50 M**	0.91	0.80	1.00
**25 M**	0.90	0.80	1.00
**10 M**	0.90	0.81	0.99
**1 M**	0.89	0.81	0.96
**0.5 M**	0.87	0.81	0.94

Spearman’s Rank Correlation was calculated between gene model expression values in synthetic read data and ‘Bowtie’-based methods for estimation of gene model expression levels. Three ‘Bowtie’-based methods for gene model expression were considered, using gene model expression values from synthetic RNAseq data of 50, 25, 10, 1, and 0.5 million total 46-mer sequence reads.

#### BowStrap runtime

Relative to BowStrap v0.5 [7], BowStrap v1.0 runs an average of 2.5 times faster, with the improvement in run time 1.2 fold for 0.5 M data set and 3.0 fold for 50 M read dataset (Table 3).

**Table 3:** Runtime of BowStrap

**Dataset**	**Total core hours**
**50 M**	35.5
**25 M**	9.1
**10 M**	6.9
**1 M**	0.8
**0.5 M**	0.2

BowStrap was used to generate gene model expression values from a synthetic RNAseq data of 50, 25, 10, 1, and 0.5 million total 46-mer sequence reads. “Total Core Hours” indicates total core hours required to perform 1000 bootstrap-style iterations. Multithread BowStrap decreases total time required approximately linearly with number of processors used, but memory required increases linearly.

## Conclusions

BowStrap, a bootstrap-style application of the ultrafast alignment program ‘Bowtie’ for estimating gene model expression from short read RNAseq datasets was introduced. BowStrap was shown to be more accurate at detecting significant gene model expression for larger datasets, and was more accurate at reporting gene model expression levels for all size datasets. Excellent accuracy of gene model expression levels even for small datasets makes this approach potentially very useful for multiplexed sequencing, allowing more samples to be run and making better use of smaller datasets. Although BowStrap does not itself identify possible splice variants in gene expression data, it can be used to highlight expressed gene models suitable to closer study, i.e. [7,11]. BowStrap 1.0 is much faster than the original BowStrap program and a multi-thread version runs even faster, run time decreasing approximately linearly with the number of processors. BowStrap is unique in its ability to assign statistical significance to gene model expression from RNAseq analysis and will be a useful addition to the set of available tools for short-read sequence analysis.

## Availability and requirements

**Project name**: BowStrap

**Project home page:**http://www.bio.anl.gov/molecular_and_systems_biology/bowstrap/bowstrap_download.html

**Operating system:** Platform independent

**Programing language:** Perl

**Other requirements:** Bowtie; Multithread Perl module for multi-processor BowStrap

**License:** GNU

**Any restrictions to use by non-academics:** None

## Abbreviations

(M): Million; (RNAseq): RNA sequencing; (NGS): Next Generation Sequencing; (RPKM): Reads Per Kill base per Million reads; (CND): Cumulative Normal Distribution.

## Competing interests

The authors declare they have no competing interests.

## Authors’ contributions

PEL designed the study. PEL and FRC wrote the manuscript. All authors read and approved the final manuscript.

## Authors’ information

The submitted manuscript has been created by UChicago Argonne LLC, Operator of Argonne National Laboratory (“Argonne”). Argonne, a U.S. Department of Energy Office of Science laboratory, is operated under Contract No. DE-AC02-06CH11357. The U.S. Government retains for itself, and others acting on its behalf, a paid-up nonexclusive, irrevocable worldwide license in said article to reproduce, prepare derivative works, distribute copies to the public, and perform publicly and display publicly, by or on behalf of the Government.
